# TPMT*3C as a Predictor of 6-Mercaptopurine-Induced Myelotoxicity in Thai Children with Acute Lymphoblastic Leukemia

**DOI:** 10.3390/jpm11080783

**Published:** 2021-08-11

**Authors:** Thawinee Jantararoungtong, Supaporn Wiwattanakul, Rawiporn Tiyasirichokchai, Santirhat Prommas, Rattanaporn Sukprasong, Napatrupron Koomdee, Pimonpan Jinda, Jiratha Rachanakul, Nutthan Nuntharadthanaphong, Samart Pakakasama, Usanarat Anurathapan, Suradej Hongeng, Chonlaphat Sukasem

**Affiliations:** 1 Division of Pharmacogenomics and Personalized Medicine, Department of Pathology, Faculty of Medicine Ramathibodi Hospital, Mahidol University, Bangkok 10400, Thailand; thawinee.jan@mahidol.ac.th (T.J.); santirhat.pro@mahidol.ac.th (S.P.); rattanaporn.suk@mahidol.ac.th (R.S.); napatruporn.kom@mahidol.ac.th (N.K.); pimonpan.jin@mahidol.ac.th (P.J.); jiratha.rac@mahidol.ac.th (J.R.); nutthan.nun@mahidol.ac.th (N.N.); 2 Laboratory for Pharmacogenomics, Somdech Phra Debaratana Medical Center (SDMC), Ramathibodi Hospital, Bangkok 10400, Thailand; 3 Department of Pathology, Faculty of Medicine, Srinakharinwirot University, Nakhon Nayok 26120, Thailand; supapowi@g.swu.ac.th (S.W.); aprilablue@hotmail.com (R.T.); 4 Division of Hematology and Oncology, Department of Pediatrics, Faculty of Medicine Ramathibodi Hospital, Mahidol University, Bangkok 10400, Thailand; samart.pak@mahidol.ac.th (S.P.); usanarat.anu@mahidol.ac.th (U.A.); suradej.hon@mahidol.ac.th (S.H.)

**Keywords:** *TPMT* genotype, 6-mercaptopurine, myelotoxicity, acute lymphoblastic leukemia

## Abstract

The response to 6-mercaptopurine (6-MP) can be altered by genetic polymorphisms in genes encoding drug-metabolizing enzymes and drug transporters. The purpose of this study was to investigate the association between genetic polymorphisms of drug-metabolizing enzymes (*TPMT 719A* > *G* (*3C), *ITPA 94C* > *A* and *ITPA 123G* > *A*) and drug transporters (*MRP4 912C* > *A* and *MRP4 2269G* > *A*) with 6-MP-related myelotoxicity and hepatotoxicity in Thai children with acute lymphoblastic leukemia (ALL). The prescribed dosage of 6-MP and its adverse effects were assessed from medical records during the first 8 weeks and 9–24 weeks of maintenance therapy. Children with the TPMT*1/*3C genotype had a higher risk of leukopenia with an odds ratio (OR) of 4.10 (95% confidence interval (CI) of 1.06–15.94; *p* = 0.033) compared to wild type (TPMT*1/*1) patients. Heterozygous TPMT*3C was significantly associated with severe neutropenia with an increased risk (OR, 4.17; 95% CI, 1.25–13.90); *p* = 0.014) during the first 8 weeks. No association was found among *ITPA* *94C* > *A, ITPA* *123G* > *A, MRP4 912C* > *A,* and *MRP4 2269G* > *A* with myelotoxicity and hepatotoxicity. The evidence that *TPMT* heterozygotes confer risks of 6-MP-induced myelotoxicity also supports the convincing need to genotype this pharmacogenetic marker before the initiation of 6-MP therapy.

## 1. Introduction

Acute lymphoblastic leukemia (ALL) is the most common hematologic malignancy diagnosed in children, and it represents 25% of all malignancies in children [1,2]. The treatment of ALL typically comprises three phases: induction of remission, intensification (or consolidation), and continuation or antimetabolite-based maintenance therapy with 6-mercaptopurine (6-MP), which is continued until two to three years from the time of diagnosis [3]. It is believed that 6-MP is the major component of increasing the cure rate. However, 6-MP-related toxicity can lead to a life-threatening situation due to the narrow therapeutic index and is the primary cause of the interruption or discontinuation of chemotherapy. In fact, between 15 and 28% of patients experience adverse drug reactions, such as myelosuppression and hepatotoxicity, when given the standard doses of 6-MP.

Pharmacogenomics holds great promise for reducing toxicity and increasing the efficacy of 6-MP in ALL treatment by assisting optimal treatment selection and appropriate dose individualization. The 6-MP treatment response can be altered by polymorphisms in genes encoding drug-metabolizing enzymes and drug transporters [4,5,6]. Thiopurine-s-methyl transferase (TPMT) catalyzes the s-methylation of 6-MP into inactive metabolite 6-methylmercaptopurine (6-MMP). Further, 6-MP is activated by hypoxanthine phosphoribosyltransferase (HPRT) and followed by a multistep metabolism to the 6-thioguanine nucleotide (6-TGN). In addition, 6-TGN is an active metabolite that can be incorporated into deoxyribonucleic acid (DNA) or ribonucleic acid (RNA) and inhibits de novo purine synthesis. Subsequently, a DNA strand will be damaged and lead to cell-cycle arrest and apoptosis.

Enzymatic activity and genetic polymorphisms of *TPMT* can influence clinical response and toxicity to 6-MP. Patients with low TPMT activity are homozygous with two variant alleles, while intermediate activity is heterozygous in the *TPMT* gene. Low and intermediate TPMT activity leads to toxicity from 6-MP, and these patients require 6-MP dose reduction. Additionally, many studies have shown significant *TPMT* polymorphism associations with myelotoxicity in children with ALL [7,8,9].

However, recent research [10,11] has shown that the genetic contribution to 6-MP toxicity is more complex, possibly involving other genes, such as inosine triphosphate pyrophosphatase (*ITPA*) and nucleoside diphosphate-linked moiety X-type motif 15 (*NUDT15*). The polymorphisms of *ITPA* that have been identified to be associated with deficiency are *ITPA 94C* > *A* and *IVS2 + 21A* > *C*. The deficiency of ITPA activity has been reported to cause the accumulation of endogenous toxic metabolites (6-thio-ITP), leading to an accumulation of methyl-thio-ITP [12]. Methyl-thio-ITP is cytotoxic and inhibits de novo purine synthesis. NUDT15 enzyme inactivates the active thiopurine metabolite thioguanosine triphosphate (TGTP) by converting TGTP to thioguanosine monophosphate (TGMP). The genetic variations in the *NUDT15* gene result in the poor metabolism of thiopurines and are associated with 6-MP toxicity [11].

Apart from drug-metabolizing enzymes, previous studies [13,14] have demonstrated that the multidrug resistance-associated protein 4 (MRP4) deficiency might experience toxicity caused by the accumulation of 6-TGN in its myeloid cells [13]. It has recently been indicated that functional gene polymorphisms of *MRP4* are a new factor attributed to thiopurine sensitivity [14].

This study aimed to investigate the frequency of drug metabolizing enzyme variants (*TPMT 719A* > *G* (*3C), *ITPA 94C* > *A* and *ITPA 123G* > *A*) and drug transporter variants (*MRP4 912 C* > *A* and *MRP4 2269G* > *A*) in Thai children with ALL and their association with 6-MP-related adverse events. 

## 2. Materials and Methods

### 2.1. Study Participants and Protocol

One hundred and fifteen Thai children with ALL, diagnosed and treated with the RAMA ALL protocol at the Division of Hematology and Oncology, Ramathibodi Hospital, Bangkok, were enrolled in this study. The ALL peripheral blood samples were classified by morphologic, cytochemistry, cytogenetic, and immunophenotypic techniques, and in some cases by molecular technology. Children were categorized into 3 risk groups (low, standard, or high) depending on pretherapeutic factors and response to induction therapy, as shown in Table 1. Patients were considered to be in the low-risk group if they were 1 to 9.9 years old presenting a white blood cell (WBC) count of less than 50 × 10^9^/L or had a DNA index of 1.16 or more. Additionally, they were excluded if they had a central nervous system (CNS) 3 status (>5 WBC/mm^3^ of cerebrospinal fluid (CSF) with blasts or cranial nerve palsy), overt testicular leukemia (documented by ultrasonographic investigation), a T-cell immunophenotype, translocation abnormality including t (9;22), t (1;19) associated with a precursor B-cell immunophenotype, a mixed lineage leukemia (*MLL*) gene rearrangement, or hypodiploid disease (<45). In poor early response, bone marrow contains 5% or more lymphoblast on Day 14 or 21 of remission induction. As for the high-risk group, the patient has t (9;22) or the breakpoint cluster region-Abelson (*BCR-ABL*) fusion gene, induction failure, or >1% leukemic blasts in the bone marrow (BM) on the remission date and >0.1% leukemic lymphoblasts in the BM in Week 7 of continuation treatment. Moreover, all cases of T-cell ALL and those of B-cell precursor ALL that did not meet the criteria for low-risk or high-risk groups were classified as a standard-risk group.

The guideline of treatment is the RAMA ALL protocol, which is modified from the total XIIIB and XV protocol of St. Jude Children’s Research Hospital (SJCRH). The details of RAMA ALL protocols have been described elsewhere [15,16]. This treatment regimen consists of three phases of therapy, namely, remission induction, consolidation, and continuation (maintenance) phase. After consolidation therapy, all of the patients received oral 6-MP-based chemotherapy plus methotrexate maintenance therapy; low-risk patients started treatment in Week 20 after diagnosis, whereas standard-risk and high-risk patients started treatment in Week 21, which continued until 2.5 years after diagnosis. Maintenance treatment differed by risk groups, as shown in Table 1. The low-risk group received 6-MP (75 mg/m^2^ daily), methotrexate (MTX, 40 mg/m^2^ weekly), vincristine (VCR, 2 mg/m^2^ monthly), and oral prednisolone (PRED, 40 mg/m^2^ 5 days/month), while the standard-risk and the high-risk groups received oral PRED 60 mg/m^2^ 5 days/month.

Additionally, the standard- and the high-risk groups received intravenous cyclophosphamide (CTX, 300 mg/m^2^ monthly) and cytarabine (Ara-C, 300 mg/m^2^ monthly). The 6-MP dose adjustment was based on the complete blood count, CBC (WBC ≥ 1500 cell/mm^3^ and absolute neutrophil count; ANC ≥ 500 cell/mm^3^), and/or infection records.

Adverse events were examined within the first six months of the initiation of maintenance treatment. Myelotoxicity was classified and graded according to the Common Terminology Criteria for Adverse Events (CTCAE) version 4.0 (https://ctep.cancer.gov/protocoldevelopment/electronic_applications/ctc.htm#ctc_40) (accessed on 1 February 2020). Myelotoxicity was defined for Grade 3 and Grade 4 as the presence of WBC (Model 1: WBC < 2000 cell/mm^3^; Model 2: WBC < 1000 cell/mm^3^), ANC (Model 1: ANC < 1000 cell/mm^3^; Model 2: ANC < 500 cell/mm^3^) and platelets (PLT) (Model 1: PLT < 50,000 cell/mm^3^; Model 2: PLT < 25,000 cell/mm^3^). Hepatotoxicity was defined when aspartate aminotransaminase (AST) and alanine aminotransaminase (ALT) were greater than 3 times the upper limit of normal.

### 2.2. Sample and Data Collection

EDTA whole blood samples were collected during continuation/maintenance chemotherapy therapy from children with ALL patients aged under 15 years old who were compliant with their 6-MP therapy and who had taken a stable dose of 6-MP for 1 month. The continuation of maintenance chemotherapy was usually administered for 2 or 3 years. The children had their full blood counts assessed at each clinic visit for the evaluation of bone marrow toxicity. The children with ALL patients who had relapse, bone marrow transplant or blood transfusion within 2 months and had a hepatic abnormality were excluded.

Clinical data, demographic data, complete blood count, and liver function tests were obtained from the medical records. The usage of 6-MP and adverse effects of the drug was assessed retrospectively from medical records. The duration of the analysis of medical records ranged from 1 to 8 weeks and from 9 to 24 weeks of maintenance therapy.

The study was conducted according to the guidelines of the Declaration of Helsinki, and approved by the Ethics Committee of Ramathibodi Hospital, Faculty of Medicine, Mahidol University (MURA2014/635 S_1_, Dec16). The study protocol was clearly explained to all participants and/or their legal guardians, and informed consent was obtained from all subjects involved in the study.

### 2.3. TPMT, ITPA, and MRP4 Genotyping

In this study, we selected single nucleotide polymorphisms (SNPs) from the CHB (Han Chinese in Beijing, China) database in the International HapMap Project (http://hapmap.ncbi.nlm.nih.gov) (accessed on 5 March 2015). The SNPs with minor allele frequencies (MAF) >5% for each gene were selected for this study. The genotyping of *TPMT 719A* > *G* (rs1142345, *3C), *ITPA 94C* > *A* (rs1127354), *ITPA* *123G* > *A* (rs13830), *MRP4 912C* > *A* (rs2274407), and *MRP4* *2269G* > *A* (rs3765534) was performed using TaqMan-based analysis on the Applied Biosystems^®^ (California, United State of America) ViiA™ 7 Real-Time polymerase chain reaction (PCR) System. The SNPs of interest were investigated in all children as well as in healthy children, but the detailed results of the healthy children are not presented in the Results section.

### 2.4. Statistical Analysis

All statistical analyses were performed using STATA10 software (Stata Corporation, College Station, TX, USA). The discrepancies between data distribution and normal distribution were examined by the Kolmogorov–Smirnov test for each group. Descriptive statistics were used to describe the clinical characteristics of the subjects. Data are expressed as the mean ± standard deviation (SD). If the variables were assumed not to be in a normal distribution, data were described as the median and interquartile range (IQR).

Genetic polymorphisms were assessed for concordance with the Hardy–Weinberg equilibrium (HWE) using Haploview 4.2. Associations among the genetic polymorphisms (alleles and genotypes), adverse events (toxicity), and clinical characteristics (age and risk group) were evaluated with the χ^2^ test, or Fisher’s exact test. The odds ratio (OR) and 95% confidence interval (CI) were calculated from the contingency table. Non-parametric tests, the Kruskal–Wallis test, and the Mann–Whitney U test were used when appropriate. Univariate logistic regression analyses were performed for the identification of factors associated with toxicity and with 6-MP metabolite levels. *p*-values < 0.05 were considered significant.

## 3. Results

### 3.1. Patient Demographics and Clinical Characteristics

The demographics and clinical characteristics of recruited patients (*n* = 115) are shown in Table 2. The mean age at diagnosis was 6.11 ± 3.86 years old. Of these patients, 63 patients were male, and 52 patients were female. Fifty-one (44.35%) patients were considered to be in the low-risk group, 50 (43.48%) in the standard-risk group, and 14 (12.17%) in the high-risk group. Since the mean age was 6.11 ± 3.86 years old, we did not find any difference in age affecting the risk groups. Although there is evidence in the literature that males may have worst clinical outcomes than females given equivalent therapy in ALL, such trends were not identified in our study.

### 3.2. Genotype and Allele Frequencies

The frequencies of variants in the *TPMT*, *ITPA*, and *MRP4* genes are shown in Table 3. The most predominant genotype of *TPMT* (rs1142345, *3C) was the homozygous wild type genotype *AA* (*n* = 102, 88.70%) followed by the heterozygous *AG* (*n* = 13, 11.30%), whereas the homozygous variant *GG* was not detected. For the *ITPA* (rs1127354) gene, 75 (65.22%) patients were carriers of the homozygous wild type genotype *CC*, 36 (31.30%) were carriers of the heterozygous *CA*, and four (3.48%) patients carried the homozygous variant *AA*. For the *ITPA* (rs13830) gene, the majority of patients were carriers of the homozygous wild type genotype *GG* (*n* = 73, 63.48%), while 38 (33.04%) patients carried heterozygous *GA*, and four (3.48%) patients carried the homozygous variant *AA*. Regarding *MRP4* (rs2274407), 88 (76.52%) patients were wild type (*CC*), and 27 (23.48%) patients were heterozygous (*CA*), with none being a homozygous carrier of the variant allele. For *MRP4* (rs3765534), the predominant genotype was the homozygous wild type genotype *GG* (*n* = 104, 90.43%) followed by the heterozygous *GA* (*n* = 11, 9.57%), whereas the homozygous variant *AA* was not detected. The risk allele frequency of *TPMT* (rs1142345, *3C) *ITPA 94C* > *A* (rs1127354), *ITPA 123G* > *A* (rs13830), *MRP4 912C* > *A* (rs2274407), and *MRP4 2269G* > *A* (rs3765534) was 0.057, 0.191, 0.200, 0.117, and 0.048, respectively. The frequency of these genotypes and alleles did not deviate from HWE (*p* > 0.05).

### 3.3. Association between Genetic Variants and 6-MP-Induced Myelotoxicity and Hepatotoxicity in the Treatment of Childhood ALL

This study aims to determine the effect of *TPMT*, *ITPA*, and *MRP4* variation in 6-MP-induced and hepatotoxicity; therefore, fifteen patients carried a genetic variant in *NUDT15* were exclude from the genetic association analyses (data not shown).

The 6-MP-induced leukopenia was observed in 49 patients during 8 weeks of maintenance therapy. When investigated by the TPMT*1/*3C genotype, the heterozygous patients (10/13; 76.92%) more frequently presented with leukopenia (WBC < 2000 cell/mm^3^) when compared with homozygous wild type patients (39/87; 44.83%) with an OR of 4.10 (95% CI, 1.06–15.95, *p* = 0.031). No significant different in other genes variants were found (Table 4). Further, 6-MP-induced neutropenia was observed in 26 patients during 8 weeks of maintenance therapy. Patients carrying the *AG* genotype of *TPMT* were significantly associated with neutropenia (ANC < 500 cell/mm^3^) compared with the *AA* genotype (OR, 4.17; 95% CI, 1.25–13.91, *p* = 0.014). There was no significant difference in other genes variants (Table 5). Further, 6-MP-induced thrombocytopenia was observed in 32 patients during 24 weeks of maintenance therapy. A significant association of thrombocytopenia (PLT < 50,000 cell/mm^3^) was identified in the patients of the *TPMT AG* genotype compared to the *AA* genotype with an OR of 4.20 (95% CI, 1.25–14.12, *p* = 0.014; Table 6). Additionally, no significant differences in other genes variants were found.

The polymorphisms at *TPMT* (rs1142345, *3C), *ITPA* (rs1127354 and rs13830), and *MRP4* (rs2274407 and rs3765534) were not significantly associated with 6-MP-induced hepatotoxicity within 8 weeks and 9–24 weeks. However, when evaluated by the TPMT*3C genotype during 8 weeks of 6-MP maintenance therapy, hepatotoxicity was more frequent in *TPMT* wild type patients (22/86; 25.58%) when compared with heterozygous TPMT*1/*3C patients. Although this statistic was not significant, the *p* value was 0.063, which nearly meets the threshold. The result is shown in Table 7.

### 3.4. Association between Genetic Variants and 6-MP Dose Intensity

The dose intensity was calculated from the ratio between 6-MP dose actually prescribed and the guideline dose. The main purpose of 6-MP dose reduction was myelotoxicity and hepatotoxicity. In this study, a significant association was identified between *MRP4 2269G* > A (rs3765534) and 6-MP dose intensity during 8 weeks and an average of 24 weeks. Patients carrying the *GA* genotype were highly sensitive to 6-MP, with a dose intensity of 52.40% at 8 weeks and an average of 24 weeks, compared with the wild type *GG* patients, who tolerated an average dose intensity of 71.45 and 66.67% at 8 weeks and an average of 24 weeks, respectively. The result is shown in Table 8.

## 4. Discussion

Pharmacogenetic studies have demonstrated the marked ethnic differences in the frequencies and the type of polymorphisms in *TPMT*, *ITPA*, and *MRP4*. This study investigated *TPMT*, *ITPA*, and *MRP4* genotypes in Thai children with ALL and analyzed the association of these genotypes and the hematological toxicity that they experienced. TPMT*3C is the most prevalent variant allele in Asian countries [17,18] and the Thai population [19,20,21,22]. For the *TPMT* gene, only the TPMT*3C variant was genotyped in our study. The allele frequency of the TPMT*3C variant was 5.7%.

This study reports the frequency of TPMT*3C in Thai children without hematologic malignancy compared to Thai children with ALL at 2.5 and 5.7%, respectively. Interestingly, most studies report a slightly higher incidence among children with ALL compared to the healthy population. A previous study from Singapore reported an incidence of 2.3% in a multiethnic migrant Asian population. The study included migrant Malay, Indian, and Chinese participants [23]. The study in Singapore reported an incidence of 7% among ALL patients. The study in Indian also reported a similar higher incidence among children with ALL compared to those without hematological malignancy (10 versus 4%) [24]. These findings may correlate with the early diagnosis of ALL if patients carry the TPMT*3C polymorphism and may be considered as a novel biomarker, but this needs further justification in future studies.

The *ITPA 94C* > *A* frequency has been studied in various populations. The frequency of allele *ITPA 94C* > *A* in this study was 19%, which is similar to the other Asian populations: 16% in Malays, 18% in Chinese, and 11% in Indians [10]. For *ITPA 123G* > *A*, our study provides the first analysis of the minor allele frequency of the “*A*” allele, which was 20%.

Our study found the allele frequency of *MRP4 2269G* > *A* and *912C* > *A* to be 4.8 and 11.7%, respectively. Our result is different from that reported in Japan, *MRP4 2269G* > *A*, *912C* > *A* (19 and 3%) [25].

This study did not find significant differences in 6-MP-induced myelotoxicity in patients carrying *ITPA 94C* > *A* (rs1127354), *ITPA 123G* > *A* (rs13830), *MRP4 912C* > *A* (rs2274407), and *MRP4* *2269G* > *A* (rs3765534) compared with the wild type. Interestingly, the statistical significance of 6-MP-induced myelotoxicity was observed in heterozygous TPMT*1/*3C after excluding the patients who had variant *NUDT15*. Patients who were heterozygous TPMT*1/*3C were significantly associated with an increased risk of 6-MP-induced leukopenia (WBC < 2000 cell/mm^3^) with an OR of 4.10 (95% CI: 1.06–15.95 (*p* = 0.031)), and 6-MP-induced neutropenia (ANC < 500 cell/mm^3^) with an OR of 4.17 (95% CI: 1.25–13.91 (*p* = 0.014)) when compared with homozygous wild type (TPMT*1/*1) patients during eight weeks of maintenance therapy. Additionally, we also found a significant association between TPMT*1/*3C and 6-MP-induced-thrombocytopenia (PLT < 25,000 cell/mm^3^) during 24 weeks of maintenance therapy when compared with TPMT*1/*1 (OR 4.20; 95% CI: 1.25–14.12; *p* = 0.014). Our findings are in concordance with previous reports that investigated an association of heterozygous genotypes of the *TPMT* gene and the myelotoxicity of drugs. Nguyen et al. reported that *TPMT* heterozygotes were associated with thiopurine-induced leukopenia (OR 4.62; 95% CI: 2.34–9.16) [26]. Additionally, almost 30–60% of patients with the heterozygous *TPMT* genotype experience severe myelosuppression when standard doses of thiopurines are administered [27].

The results of this study agree with previous reports that investigated an association of heterozygous genotypes of the *TPMT* gene and the myelotoxicity of drugs. Our study showed that 6-MP therapy is associated with increased myelotoxicity during maintenance therapy. This is due to the fact that the low activity of TPMT caused by the TPMT*3C genetic variant could not process the prodrug 6-MP properly, and the concentration of this drug raises over time in the blood and produces myelotoxicity.

*TPMT* genotyping was not performed before the initial dose adjustment in the RAMA ALL protocol. Although there was a statistically significant correlation with myelotoxicity in patients carrying the *TPMT* heterozygous genotype (*1/*3C), this study suggests an urgent dose adjustment in these patients. However, from the medical records, we found that dose was not adjusted probably as the patients had infection, and there was also no median 6-MP dose difference in either heterozygous or wild type patients. Although no dose difference was identified from the medical records of the patients carrying either the heterozygous (*1/*3C) or wild type (*1/*1) genotype of *TPMT*, since myelotoxicity was significantly associated with patients heterozygous for the genetic polymorphism of *TPMT* gene, this study suggests the careful management of 6-MP therapy in ALL patients carrying this variant.

The median of dose intensity of patients with heterozygous *MRP4 2269G* > *A* was significantly lower than that of wild type patients during eight weeks of maintenance therapy (*p* = 0.006) and an average of 24 weeks of maintenance therapy (*p* = 0.020). Therefore, *MRP4 2269G* > *A* may be useful for personalizing the therapeutic dose of 6-MP during maintenance therapy in Thai ALL patients. This finding is in concordance with a previous study [25], which reported a dose reduction in patients who carried homozygous *MRP4 2269G* > *A* compared with wild type (*p* = 0.024).

Hepatoxicity is a common adverse drug reaction during maintenance therapy with 6-MP, resulting in elevated levels of liver enzymes. Although not statistically significant, patients with wild type *TPMT* developed more severe hepatotoxicity with high levels of ALT for eight weeks of maintenance therapy compared with TPMT*1/*3C. Our result agrees with that of the previous study [28,29], showing the higher ALT levels in wild type *TPMT*. Relling et al. [29] also found no evidence of hepatotoxicity in homozygous and heterozygous *TPMT* variants. Hepatotoxicity was found to be more frequent among those with high TPMT activity. This result could be explained by the hepatotoxic effects of 6-thioinosine triphosphate (6-MeTITP). Further, 6-MeTITP inhibits de novo purine synthesis, and thioinosine triphosphate (MeTITP) might be toxic with hepatic cells. The previous study [8] showed the association between ALT and MeTITP. Consequently, low ALT levels might reveal the poor adherence or reduced bioavailability of 6-MP, at least within the *TPMT* wild type patients.

Maintenance therapy is as important as the more intensive and toxic earlier treatment phases, and often more challenging. Therefore, prospective studies analyzing the involvement of *TPMT*, *ITPA*, and *MRP4* polymorphisms and adverse reactions to 6-MP are necessary. The measurement of active 6-MP metabolite concentrations must be performed complementarily to genotyping in predicting toxicity under treatment with 6-MP. Continuing research should address the applicability of drug metabolite measurements for dose adjustments, extensive host genome profiling to understand diversity in treatment efficacy and toxicity, and alternative thiopurine dosing regimens to improve therapy for the individual patient. Moreover, 6-TG-DNA is a DNA-incorporated thioguanine nucleotide (DNA-TGN). In the study of Nielsen et al. [30], they measured 6-TG-DNA concentrations in ALL patents from blood leukocytes during maintenance therapy and found that they are associated with relapse-free survival (RFS). Moreover, the adjusted dose to maintain higher DNA-TGN levels might reduce the relapse rate, which is a benefit for monitoring the thiopurine efficacy. However, the monitoring of 6-TG-DNA with thiopurine toxicity was not elucidatory. The measurement of mercaptopurine metabolites might be suitable for routine monitoring.

Lastly, our previous study [11] showed that *NUDT15* variants may cause neutropenia, and that the 6-MP dosage should be considered in patients according to the *NUDT15* variants to inform personalized 6-MP therapy in pediatric patients. The *NUDT15* variants were associated with neutropenia as compared with wild type genotype (OR 17.862, 95% CI: 4.198–75.992, *p* = 9.5 × 10^−5^). However, in this study, we excluded patients who carried the *NUDT15* variant from the genetic analysis. We, therefore, could not determine the effect of both genes on the adverse effect of 6-MP in this population.

## 5. Conclusions

TPMT*3C was found to be significantly associated with the development of 6-MP-induced myelotoxicity in Thai pediatric ALL patients. Since the Food and Drug Administration (FDA) drug label recommends *TPMT* testing in patients taking 6-MP who experience myelotoxicity, the findings of this study also support this recommendation. The pharmacogenetic testing of *TPMT* in ALL patients taking 6-MP may reduce myelotoxicity in a considerable proportion of patients.

## Figures and Tables

**Table 1:** Risk classification of RAMA ALL protocol and chemotherapy in each risk group.

Risk Classification	RAMA ALL Protocol Details	Chemotherapy in Maintenance Phase
Low	Precursor B-cell ALL with DNA index > 1.16, or age 1 to 9.9 years, WBC count < 50 × 10^9^/L Must not have - CNS 3 status - Overt testicular leukemia - Adverse genetic features [t (9;22), t (1;19), *MLL* or hypodiploid disease (<45)] - Poor early response (lymphoblast ≥ 5% on Day 14 or 21)	6-MP 75 mg/m^2^ PO daily MTX 40 mg/m^2^ PO weekly VCR 2 mg/m^2^ IVP monthly PRED 40 mg/m^2^ PO 5 days/month
Standard	All cases of T-cell ALL and those of B-cell precursor ALL that do not meet the criteria for low risk or high-risk group	6-MP 75 mg/m^2^ PO daily MTX 40 mg/m^2^ PO weekly VCR 2 mg/m^2^ IVP monthly PRED 60 mg/m^2^ PO 5 days/month CTX 300 mg/m^2^ IV monthly Ara-C 300 mg/m^2^ IV monthly
High	t(9;22) or *BCR-ABL* fusion gene Induction failure or >1% leukemic blasts in the BM on remission date >0.1% leukemic lymphoblast in the BM in Week 7 of continuation treatment	

Abbreviations: ALL, acute lymphoblastic leukemia; DNA, deoxyribonucleic acid; WBC, white blood cell; CNS, central nervous system; *MLL*, mixed lineage leukemia; *BCR-ABL*, breakpoint cluster region-Abelson fusion gene; BM, bone marrow; 6-MP, 6-mercaptopurine; MTX, methotrexate; VCR, vincristine; PRED, prednisolone; CTX, cyclophosphamide; Ara-C, cytarabine.

**Table 2:** Demographics and clinical characteristics of recruited patients (*n* = 115).

Characteristics	*n*
Age at diagnosis, years (mean ± SD)	6.11 ± 3.86
Gender (*n*, %)	
Male	63 (54.78)
Female	52 (45.22)
Risk group (*n*, %)	
High	14 (12.17)
Standard	50 (43.48)
Low	51 (44.35)

Abbreviations: SD, standard deviation; *n* = number of samples in the analysis.

**Table 3:** Genotype and allele frequencies of *TPMT*, *ITPA*, and *MRP4* variants (*n* = 115).

Genes/SNPs	Genotype	Genotype Frequency *n* (%)	MAF	*p*-Value
*TPMT 719A* > *G* (rs1142345, *3C)	*AA* (*1/*1) *AG* (*1/*3C)	102 (88.70) 13 (11.30)	*G* = 0.057	1.000
*ITPA 94C* > *A* (rs1127354)	*CC* *CA* *AA*	75 (65.22) 36 (31.30) 4 (3.48)	*A* = 0.191	1.000
*ITPA 123G* > *A* (rs13830)	*GG* *GA* *AA*	73 (63.48) 38 (33.04) 4 (3.48)	*A* = 0.200	1.000
*MRP4 912C* > *A* (rs2274407)	*CC* *CA*	88 (76.52) 27 (23.48)	*A* = 0.117	0.355
*MRP4 2269G* > *A* (rs3765534)	*GG* *GA*	104 (90.43) 11 (9.57)	*A* = 0.048	1.000

Abbreviations: *TPMT*, thiopurine-s-methyl transferase; *ITPA*, inosine triphosphate pyrophosphatase; *MRP4*, multidrug resistance-associated protein 4; SNPs, single nucleotide polymorphisms; MAF, minor allele frequency; *n* = number of samples in the analysis.

**Table 4:** The association of *TPMT*, *ITPA*, and *MRP4* genetic polymorphisms with leukopenia during treatment with 6-MP (*n* = 100).

Gene/SNPs	Genotype *n* (%)	WBC Weeks 1–8						WBC Weeks 9–24					
		Model 1 (WBC < 2000 cell/mm^3^)			Model 2 (WBC < 1000 cell/mm^3^)			Model 1 (WBC < 2000 cell/mm^3^)			Model 2 (WBC < 1000 cell/mm^3^)		
		Toxic	Non-Toxic	*p*-Value	Toxic	Non-Toxic	*p*-Value	Toxic	Non-Toxic	*p*-Value	Toxic	Non-Toxic	*p*-Value
TPMT*3C 719A > G													
*AA* (*1/*1)	87 (87)	39 (44.83)	48 (55.17)	**0.031**	6 (6.90)	81 (93.10)	1.000	58 (66.67)	29 (33.33)	0.101	19 (21.84)	68 (78.16)	1.000
*AG* (*1/*3C)	13 (13)	10 (76.92)	3 (23.08)		1 (7.69)	12 (92.31)		12 (92.31)	1 (7.69)		2 (15.38)	11 (84.62)	
***ITPA 94C* > *A***													
*CC*	65 (65)	33 (51.52)	32 (48.48)	0.814	4 (6.06)	61 (93.94)	0.760	45 (68.18)	20 (31.82)	1.000	11 (16.67)	54 (83.33)	0.325
*CA*	31 (31)	13 (41.94)	18 (58.06)		3 (9.67)	28 (90.32)		22 (70.96)	9 (29.03)		9 (29.03)	22 (70.96)	
*AA*	4 (4)	2 (50)	2 (50)		0 (0)	4 (100)		3 (75)	1 (25)		0 (0)	4 (100)	
***ITPA 123G* > *A***													
*GG*	63 (63)	31 (49.20)	32 (50.80)	0.935	3 (4.84)	60 (95.16)	0.425	43 (67.74)	20 (32.26)	0.923	12 (19.35)	51 (80.65)	0.668
*GA*	33 (33)	15 (45.45)	18 (54.54)		4 (12.12)	29 (87.87)		24 (72.72)	9 (27.27)		8 (24.24)	25 (75.75)	
*AA*	4 (4)	2 (50)	2 (50)		0 (0)	4 (100)		3 (75)	1 (25)		0 (0)	4 (100)	
***MRP4 912 C* > *A***													
*CC*	80 (80)	40 (50)	40 (50)	0.764	7 (8.75)	73 (91.25)	0.339	56 (70)	24 (30)	1.000	19 (23.75)	61 (76.25)	0.348
*CA*	20 (20)	9 (45)	11 (55)		0 (0)	20 (100)		14 (70)	6 (30)		2 (10)	18 (90)	
***MRP4 2269G* > *A***													
*GG*	92 (92)	46 (50)	46 (50)	0.717	7 (7.61)	85 (92.39)	1.000	66 (71.74)	26 (28.26)	0.236	20 (21.74)	72 (78.26)	1.000
*GA*	8 (8)	3 (37.50)	5 (62.50)		0 (0)	8 (100)		4 (50)	4 (50)		1 (12.50)	7 (87.50)	

Abbreviations: SNPs, single nucleotide polymorphisms; *TPMT*, thiopurine-s-methyl transferase; *ITPA*, inosine triphosphate pyrophosphatase; *MRP4*, multidrug resistance-associated protein 4; WBC, white blood cell; *n* = number of samples in the analysis. Bold values denote statistical significance at the *p* < 0.05 level.

**Table 5:** The association of *TPMT*, *ITPA*, and *MRP4* genetic polymorphisms with neutropenia during 6-MP treatment (*n* = 100).

Gene/SNPs	Genotype *n* (%)	ANC Weeks 1–8						ANC Weeks 9–24					
		Model 1 (ANC < 1000 cell/mm^3^)			Model 2 (ANC < 500 cell/mm^3^)			Model 1 (ANC < 1000 cell/mm^3^)			Model 2 (ANC < 500 cell/mm^3^)		
		Toxic	Non-Toxic	*p*-Value	Toxic	Non-Toxic	*p*-Value	Toxic	Non-Toxic	*p*-Value	Toxic	Non-Toxic	*p*-Value
TPMT*3C 719A > *G*													
*AA* (*1/*1)	87 (87)	49 (56.32)	38 (43.68)	0.549	19 (21.84)	68 (78.16)	**0.014**	57 (65.52)	30 (34.48)	0.215	40 (45.98)	47 (54.02)	0.543
*AG* (*1/*3C)	13 (13)	9 (69.23)	4 (30.77)		7 (53.85)	6 (46.15)		11 (84.62)	2 (15.38)		7 (53.85)	6 (46.15)	
***ITPA 94C* > *A***													
*CC*	65 (65)	40 (60.61)	25 (39.39)	0.354	16 (25.76)	49 (74.27)	0.590	44 (66.67)	21 (33.33)	1.000	30 (46.97)	35 (53.03)	0.816
*CA*	31 (31)	18 (58.06)	14 (41.93)		9 (29.03)	22 (70.96)		21 (67.74)	10 (32.26)		15 (48.38)	16 (51.62)	
*AA*	4 (4)	3 (75)	1 (25)		0 (0)	4 (100)		3 (75)	1 (25)		1 (25)	3 (75)	
***ITPA 123G* > *A***													
*GG*	63 (63)	39 (61.29)	24 (38.71)	0.314	15 (24.19)	47 (75.81)	0.546	43 (67.74)	20 (32.26)	1.000	30 (48.39)	33 (51.61)	0.818
*GA*	33 (33)	18 (54.54)	15 (45.45)		10 (30.30)	23 (69.69)		22 (66.67)	11 (33.33)		15 (45.45)	18 (54.54)	
*AA*	4 (4)	1 (25)	3 (75)		0 (0)	4 (100)		3 (75)	1 (25)		1 (25)	3 (75)	
***MRP4 912 C* > *A***													
*CC*	80 (80)	54 (58.70)	38 (41.30)	0.213	23 (25)	69 (75)	0.387	52 (65)	28 (35)	0.186	35 (43.75)	45 (56.25)	0.322
*CA*	20 (20)	4 (50)	4 (50)		3 (37.50)	5 (62.50)		11 (55)	9 (45)		8 (40)	12 (60)	
***MRP4 2269G* > *A***													
*GG*	92 (92)	54 (93.10)	38 (90.48)	0.717	22 (88)	70 (93.33)	0.409	64 (69.57)	28 (30.43)	0.264	45 (48.91)	47 (51.09)	0.282
*GA*	8 (8)	4 (6.9)	4 (9.52)		3 (12)	5 (6.67)		4 (50)	4 (50)		2 (25)	6 (75)	

Abbreviations: SNPs, single nucleotide polymorphisms; *TPMT*, thiopurine-s-methyl transferase; *ITPA*, inosine triphosphate pyrophosphatase; *MRP4*, multidrug resistance-associated protein 4; ANC, absolute neutrophil count; *n* = number of samples in the analysis. Bold values denote statistical significance at the *p* < 0.05 level.

**Table 6:** The association of *TPMT*, *ITPA*, and *MRP4* genetic polymorphisms with thrombocytopenia during treatment with 6-MP (*n* = 100).

Gene/SNPs	Genotype *n* (%)	PLT Weeks 1–8						PLT Weeks 9–24					
		Model 1 (PLT < 50,000 cell/mm^3^)			Model 2 (PLT < 25,000 cell/mm^3^)			Model 1 (PLT < 50,000 cell/mm^3^)			Model 2 (PLT < 25,000 cell/mm^3^)		
		Toxic	Non-Toxic	*p*-Value	Toxic	Non-Toxic	*p*-Value	Toxic	Non-Toxic	*p*-Value	Toxic	Non-Toxic	*p*-Value
TPMT*3C 719A > *G*													
*AA* (*1/*1)	87 (87)	6 (6.90)	81 (93.10)	1.000	3 (3.45)	84 (96.55)	0.432	24 (27.59)	63 (72.41)	**0.014**	3 (3.45)	84 (96.55)	0.432
*AG* (*1/*3C)	13 (13)	1 (7.69)	12 (92.31)		1 (7.69)	12 (92.31)		8 (61.54)	5 (38.46)		1 (7.69)	12 (92.31)	
***ITPA 94C* > *A***													
*CC*	65 (65)	7 (10.61)	58 (89.39)	0.132	3 (4.55)	62 (95.45)	1.000	20 (30.30)	45 (69.70)	0.789	2 (3.03)	63 (96.97)	0.655
*CA*	31 (31)	0 (0)	31 (100)		1 (2.94)	30 (97.06)		10 (35.29)	21 (64.71)		2 (5.88)	29 (94.12)	
*AA*	4 (4)	0 (0)	4 (100)		0 (0)	4 (100)		2 (50)	2 (50)		0 (0)	4 (100)	
***ITPA 123G* > *A***													
*GG*	63 (63)	7 (11.29)	56 (88.71)	0.133	3 (4.84)	60 (95.16)	1.000	19 (30.65)	44 (69.35)	0.685	2 (3.23)	61 (94.74)	0.666
*GA*	33 (33)	0 (0)	33 (100)		1 (2.63)	32 (97.37)		11 (34.21)	22 (65.79)		2 (50)	31 (32.29)	
*AA*	4 (4)	0 (0)	4 (100)		0 (0)	4 (100)		2 (50)	2 (50)		0 (0)	4 (100)	
***MRP4 912 C* > *A***													
*CC*	80 (80)	7 (8.75)	73 (91.25)	0.339	4 (5)	76 (95)	0.581	28 (35)	52 (65)	0.285	3 (3.75)	77 (96.25)	1.000
*CA*	20 (20)	0 (0)	20 (100)		0 (0)	20 (100)		4 (20)	16 (80)		1 (5)	19 (95)	
***MRP4 2269G* > *A***													
*GG*	92 (92)	7 (7.61)	85 (92.39)	1.000	4 (4.35)	88 (95.65)	1.000	30 (32.61)	62 (67.39)	1.000	3 (3.26)	89 (96.74)	0.287
*GA*	8 (8)	0 (0)	8 (100)		0 (0)	8 (100)		2 (25)	6 (75)		1 (12.50)	7 (87.50)	

Abbreviations: SNPs, single nucleotide polymorphisms; *TPMT*, thiopurine-s-methyl transferase; *ITPA*, inosine triphosphate pyrophosphatase; *MRP4*, multidrug resistance-associated protein 4; PLT, platelets; *n* = number of samples in the analysis. Bold values denote statistical significance at the *p* < 0.05 level.

**Table 7:** The association of *TPMT, ITPA,* and *MRP4* genetic polymorphisms with hepatotoxicity: AST/ALT (during treatment with 6-MP) (*n* = 98).

Gene/SNPs	Genotype	Liver Weeks 1–8						Liver Weeks 9–24					
	*n* (%)	AST			ALT			AST			ALT		
		Toxic	Non-Toxic	*p*-Value	Toxic	Non-Toxic	*p*-Value	Toxic	Non-Toxic	*p*-Value	Toxic	Non-Toxic	*p*-Value
TPMT*3C 719A > *G*													
*AA* (*1/*1)	86 (87.75)	9 (10.47)	77 (89.53)	1.000	22 (25.58)	64 (72.42)	0.063	19 (22.09)	67 (77.91)	0.115	28 (32.56)	58 (67.44)	1.000
*AG* (*1/*3C)	12 (12.25)	1 (8.33)	11 (91.67)		0 (0)	12 (100)		0 (0)	12 (100)		4 (33.33)	8 (66.67)	
***ITPA 94C* > *A***													
*CC*	64 (65.30)	9 (14.06)	55 (85.94)	0.158	15 (23.44)	49 (76.56)	0.805	15 (23.44)	49 (76.56)	0.191	21 (32.81)	43 (67.19)	1.000
*CA + AA*	34 (34.70)	1 (2.94)	33 (97.05)		7 (20.59)	27 (79.41)		4 (11.76)	30 (88.24)		11 (32.35)	23 (67.65)	
***ITPA 123G* > *A***													
*GG*	62 (63.26)	9 (14.52)	53 (85.48)	0.087	14 (22.58)	48 (77.42)	1.000	15 (24.19)	47 (75.81)	0.184	20 (32.26)	42 (67.74)	1.000
*GA + AA*	36 (36.74)	1 (2.78)	35 (97.22)		8 (22.22)	28 (77.78)		4 (11.11)	32 (88.89)		12 (33.33)	24 (66.67)	
***MRP4_C912 C* > *A***													
*CC*	78 (79.59)	9 (11.54)	69 (88.46)	0.682	17 (21.79)	61 (78.21)	0.768	13 (16.67)	65 (83.33)	0.209	24 (30.77)	54 (69.23)	0.436
*CA*	20 (20.41)	1 (5)	19 (95)		5 (25)	15 (75)		6 (30)	14 (70)		8 (40)	12 (60)	
***MRP4_2269G* > *A***													
*GG*	91 (92.85)	10 (10.99)	81 (89.01)	1.000	19 (20.88)	72 (79.12)	0.186	18 (19.78)	73 (80.22)	1.000	31 (34.07)	60 (65.93)	0.421
*GA*	7 (7.15)	0 (0)	7 (100)		3 (42.85)	4 (5.25)		1 (14.29)	6 (85.71)		1 (14.29)	6 (85.71)	

Abbreviations: SNPs, single nucleotide polymorphisms; *TPMT*, thiopurine-s-methyl transferase; *ITPA*, inosine triphosphate pyrophosphatase; *MRP4*, multidrug resistance-associated protein 4; AST, aspartate aminotransaminase; ALT, alanine aminotransaminase; *n* = number of samples in the analysis. Bold values denote statistical significance at the *p* < 0.05 level.

**Table 8:** Association between genetic polymorphism and 6-MP dose intensity at 8 weeks, 9–24 weeks, and an average of 24 weeks (*n* = 115).

Gene/SNPs	*n* (%)	8 Weeks (Dose Intensity, %)	*p*-Value	9–24 Weeks (Dose Intensity, %)	*p*-Value	An Average of 24 Weeks (Dose Intensity, %)	*p*-Value
TPMT*3C							
*AA* (*1/*1)	102 (88.69)	66.67 (57.07–100)	0.852	66.67 (47.60–85.63)	0.641	66.67 (52.40–88.09)	0.754
*AG* (*1/*3C)	13 (11.31)	66.67 (47.61–100)		66.67 (41.68–83.33)		66.67 (44.40–91.66)	
***ITPA_94C* > *A***							
*CC*	75 (65.22)	66.67 (57.07–98.8)	0.882	66.07 (47.60–85.69)	0.783	66.67 (52.40–87.50)	0.800
*AC*	36 (31.30)	66.67 (52.40–109)		66.67 (47.60–76.13)		66.67 (52.27–90.40)	
*AA*	4 (3.48)	83.33 (63.07–100)		69.0 (55.94–92.83)		76.17 (59.50–96.41)	
***ITPA_123G* > *A***							
*GG*	73 (63.48)	66.67 (57.07–97.60)	0.882	66.67 (47.60–85.73)	0.783	66.67 (52.36–86.91)	0.800
*GA*	38 (33.04)	66.67 (52.40–110)		66.67 (47.60–76.03)		66.67 (52.35–88.66)	
*AA*	4 (3.48)	89.0 (63.07–100)		89.0 (55.94–92.8)		76.17 (59.30–9.641)	
***MRP4_912C* > *T***							
*CC*	88 (76.52)	69.0 (57.07–100)	0.593	66.67 (52.43–89.82)	0.902	66.67 (47.60–85.06)	0.830
*CT*	27 (23.48)	66.67 (52.40–100)		66.63 (66.03–83.33)		66.67 (47.60–85.60)	
***MRP4_2269G* > *A***							
*GG*	104 (90.43)	71.45 (61.87–133)	**0.006**	66.67 (47.60–85.73)	0.074	66.67 (54.73–91.24)	**0.020**
*GA*	11 (9.57)	52.40 (47.60–85.73)		47.60 (38.09–66.67)		52.40 (45.25–71.43)	

Abbreviations: SNPs, single nucleotide polymorphisms; *TPMT*, thiopurine-s-methyl transferase; *ITPA*, inosine triphosphate pyrophosphatase; *MRP4*, multidrug resistance-associated protein 4; *n* = number of samples in the analysis. Dose intensity was calculated by dividing the 6-MP dose actually prescribed by the guideline dose and multiplying by 100. Bold values denote statistical significance at the *p* < 0.05 level.
